# Does gamma-aminobutyric acid (GABA) influence the development of chronic inflammation in rheumatoid arthritis?

**DOI:** 10.1186/1742-2094-5-1

**Published:** 2008-01-03

**Authors:** James M Kelley, Laura B Hughes, S Louis Bridges

**Affiliations:** 1 Division of Clinical Immunology and Rheumatology, Department of Medicine, University of Alabama at Birmingham, Birmingham, Alabama, USA

## Abstract

**Background:**

Recent studies have demonstrated a role for spinal p38 MAP kinase (MAPK) in the development of chronic inflammation and peripheral arthritis and a role for GABA in the inhibition of p38 MAPK mediated effects. Integrating these data suggests that GABA may play a role in downregulating mechanisms that lead to the production of proinflammatory agents such as interleukin-1, interleukin-6, and matrix metalloproteinase 3 – agents implicated in the pathogenesis of rheumatoid arthritis (RA). Genetic studies have also associated RA with members of the p38 MAPK pathway.

**Hypothesis:**

We propose a hypothesis for an inefficient GABA signaling system that results in unchecked proinflammatory cytokine production via the p38 MAPK pathway. This model also supports the need for increasing research in the integration of immunology and neuroscience.

## Background

The impact of an immune response on the nervous system has long been apparent, with multiple sclerosis (MS), myasthenia gravis (MG), and neuropsychiatric manifestations of systemic lupus erythematosus serving as examples. While neuroendocrine modulation of the immune system has been appreciated, the influence of the nervous system on immune responses is not well understood. The complex characters of a coordinated immune response and the nervous/endocrine (electrical/chemical) communication systems of the body complicate research in this area; however, relationships between the nervous and immune systems are essential for proper function. Any entity would be compromised if its defense and communication systems did not interact – humans are no exception.

Rheumatoid arthritis (RA) is a common systemic inflammatory condition leading to symmetric, chronic synovial inflammation, erosions, and joint destruction in some patients. Its etiology is unclear but may result from an environmental trigger in the context of genetic predisposition. There is literature precedent for neuroendocrine involvement in RA pathogenesis [1] including suggestions of neurotransmitters such as norepinephrine impacting RA [2] and suggesting a role for the hypothalamic-pituitary-adrenal axis in inflammation [3].

A recent article reported that inhibiting spinal cord p38 MAP kinase (MAPK) reduced joint inflammation in the rat model of RA by an unknown mechanism [4]. Somatic afferent pain signals received in the spinal cord result in stress-induced kinase release, causing efferent signals that direct mediators of inflammatory response. Spinal p38 MAPK inhibition lowered levels of the pro-inflammatory mediators, interleukin 1 (IL-1), interleukin 6 (IL-6), and matrix metalloproteinase 3 (MMP3), in the periphery, thereby linking the central nervous system (CNS) with peripheral immune responses [4]. IL-1 also functions as a neuroendocrine modulator in animal models of RA [5], as a cytokine contributing to joint destruction [6], and as a product of pain facilitation responses of spinal cord glial cells [7].

Proinflammatory cytokine production via p38 MAPK appears to be dependent on tumor necrosis factor-alpha (TNF-α) since administration of etanercept (TNF inhibitor) blocks proinflammatory effects in rats [4]. If spinal administration of a TNF inhibitor is effective in reducing inflammatory disease with less systemic side-effects [8], it may be an appropriate, albeit more cumbersome, delivery method for this class of treatment.

p38 MAPK is an important intermediate in prostaglandin release. Prostaglandins can modulate an inflammatory response and sensitize neurons to pain. Activation of spinal N-methyl-D-aspartic acid (NMDA) receptors results in release of prostaglandins and mediators of thermal hyperalgesia. p38 MAPK inhibition downregulates this process [9], providing another example of immune regulation by neuronal p38 MAPK. p38 MAPK is encoded by *MAPK14 *on Chromosome 6p21.3. Its alpha and gamma isoforms are implicated in pathways leading to chronic inflammation [10]. p38 MAPK activates and interacts with immunomodulators such as signal transducer and activator of transcription 4 (*STAT4*), which has been recently associated with susceptibility to RA [11], other map kinase molecules (such as ERKs and MKKs), and NFκB. p38 MAPK, TNF, NFκB, and STAT4 are potential pharmacological targets; therefore, research is intensifying to detect subtleties of this pathway on inflammatory disease. One subtlety that should be examined in this research is determining if neurotransmitters participate in RA pathogenesis.

## Hypothesis

We propose a model in which gamma-aminobutyric acid (GABA), the primary inhibitory neurotransmitter of the CNS, may downregulate p38 MAPK activity to reduce peripheral production of proinflammatory cytokines in joints affected by RA.

Afferent pain signals contribute to the CNS propagating an inflammatory response that may influence the development of peripheral arthritis [4]. Crosstalk between the immune and nervous systems can inhibit p38 MAPK and may downregulate a chronic inflammatory response. GABA prevents release of IL-6 by inhibiting p38 MAPK in rat glioma cells [12]. While the effects of GABA signaling on release of other proinflammatory cytokines such as IL-1 and TNF-α have not been examined, IL-1 is known to impact GABA function [13].

GABA may reduce inflammation, or, conversely, deficient GABA function may contribute to uncontrolled inflammation. Such deficient function could likely occur at a specific GABA receptor. GABA_B _receptors are metabotropic and produce prolonged inhibitory signals, which make a more likely candidate for impacting a chronic inflammatory response. They differ from ionotropic GABA receptors that utilize fast synaptic transmission. The GABA_B _receptor is a heterodimer with subunits encoded by *GABBR1 *and *GABBR2*. *GABBR1 *is encoded in the Major Histocompatibility Complex (MHC) extended class I region (6p21.3) [14], an area of the MHC associated with MS, MG, Alzheimer's disease, schizophrenia, narcolepsy, epilepsy, and RA. While specific *HLA-DRB1 *alleles (MHC class II) contribute strongly to RA susceptibility, possibly due to their role in presenting arthritogenic peptides, *GABBR1 *polymorphisms are not in linkage disequilibrium with these alleles (within publicly available HapMap data) as is expected given the 3 Mb distance between the two loci. Thus, observed genetic association to this region suggests a potentially independent role for *GABBR1 *in genetic susceptibility to RA. *GABBR1 *polymorphisms have not been experimentally characterized in RA patients but computational analyses suggest that some may influence alternative splicing [15] or protein structure [16]. *GABBR1 *encodes multiple isoforms and missense mutations [17] that are implicated in other disease processes [18] further supporting this possibility.

Since *GABBR1 *and *MAPK14 *are both encoded on 6p21.3, their expression may be regulated together, which is common for functionally related genes in or near the MHC [19,20]. Since GABA, which may downregulate inflammation, requires a receptor to signal, a *GABBR1 *polymorphism may impair downstream inhibition of p38 MAPK. Other molecules are known to influence p38 MAPK function in an arthritic state, including map kinase family members (such as ERK1/2) and NFκB [21], and likely also participate in these complex interactions of RA pathogenesis.

**Figure 1:**
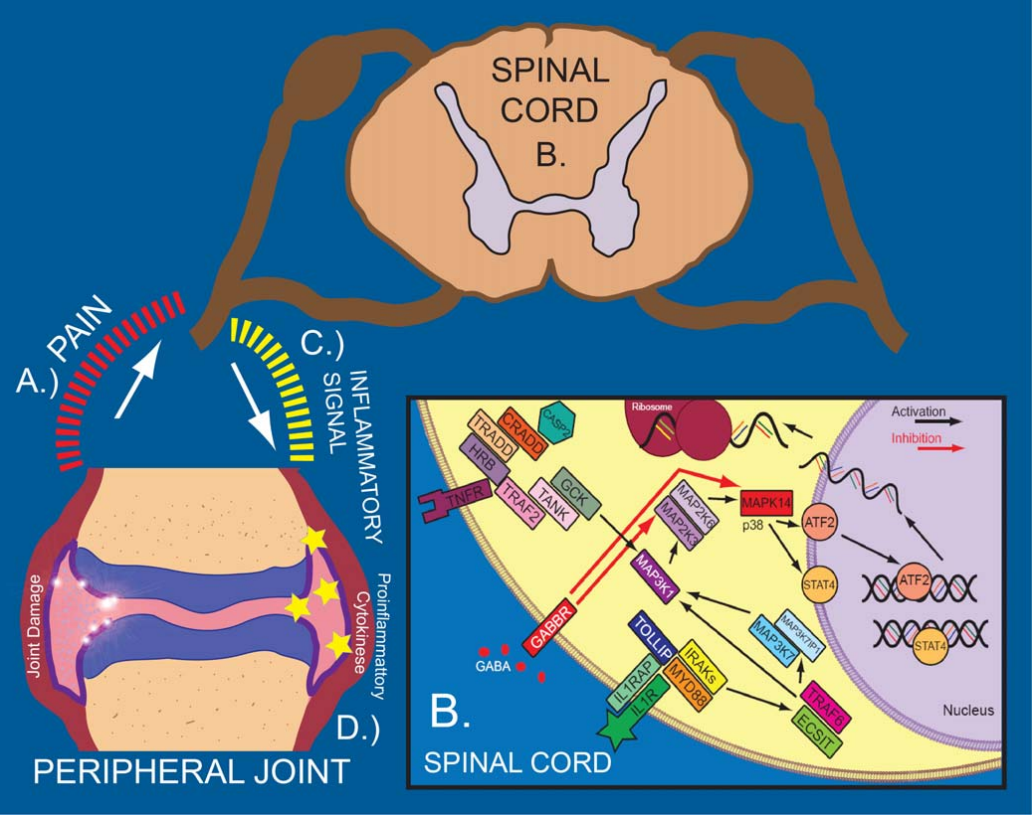
**Possible influence of GABA on p38 MAPK pathway in rheumatoid arthritis**. This figure illustrates a possible model for how GABA can inhibit the production of proinflammatory cytokines. All molecules are listed by their official gene name.

Within that complex system, we propose a potential mechanism that may contribute to chronic inflammation in RA (Figure 1). A.) When synovial destruction begins, afferent somatic fibers carry nociceptive impulses to the spinal cord. B.) In the spinal cord, these pain and/or cytokine signals activate p38 MAPK. C.) Spinal p38 MAPK which then interacts with STAT4 or other molecules to induce signals that upregulate additional proinflammatory cytokine release such as IL-1, IL-6, or MMP3 in the periphery [4,7]. (While the link between spinal cord receptor signaling and peripheral immune responses is not fully understood, there is precedent for such relationships [22]. For example, inhibited p38 MAPK signaling in the spinal cord can trigger acetylcholine release in the periphery [23].) D.) These proinflammatory molecules produce additional joint destruction and excessive immune response.

There are mechanisms that can negatively regulate this pathway. An inhibitory signal to the spinal cord (ex. GABA) may downregulate p38 MAPK and limit proinflammatory cytokine production [12]. This could reduce an exacerbation of RA resulting from additional proinflammatory cytokines. Any variation affecting this negative regulation, such as a SNP allele or haplotype in *GABBR1*, may allow p38 MAPK to proceed unchecked and worsen RA.

Recent genetic evidence and functional studies in animals suggest a neurological component to RA pathogenesis and need for additional study. More insight into RA pathogenesis could also improve understanding of neurological conditions. Proinflammatory cytokines, which exacerbate synovial damage in RA, are present in excessive levels in neurodegenerative diseases [24]. Understanding this excess cytokine production in RA, or in neurodegeneration, may assist research and ultimately treatment for the other condition. One of these excessive proinflammatory cytokines, IL-6, is implicated in improper beta-amyloid processing during Alzheimer's disease (AD) due to its effects on alpha-2-macroglobulin (*A2M*) [25]. A2M is a protease inhibitor that clears beta-amyloid components; when not functioning properly, plaques accumulate contributing to AD [26]. A2M is also a cytokine transporter that is overly consumed in synovial fluid of RA patients [27], causing an accumulation of materials (cytokines) and contributing to disease. These two pathogenic mechanisms use the same molecule (A2M) in a similar manner (resulting in accumulation of materials): studying the mechanism in one disease may help understanding pathogenesis of the other disease.

## Implications

In summary, inhibition of p38 MAPK can limit joint inflammation [4], and GABA can reduce p38 MAPK signaling [12]. However, no direct link has been reported between GABA and joint inflammation. We hypothesize that GABA may influence RA pathogenesis via the p38 MAPK pathway as depicted in Figure 1.

One difficulty in establishing these links (i.e. relating GABA to immune system physiology) is assembling the resources and expertise needed to evaluate a neuroimmunological question. While many researchers are focused on neuroscience or immunology, few laboratories have the experience to combine an understanding of neuronal/glial molecular pathways and rheumatic/autoimmune diseases. Furthermore, researchers in these areas do not frequently collaborate. We would encourage additional work on the role of spinal cord receptor signaling on the development of RA, and we hope that suggesting a role for GABA in the development of RA will not only encourage the further integration of basic immunology into clinical neuroscience but encourage the incorporation of basic neuroscience into clinical immunology.

## Competing interests

The author(s) declare that they have no competing interests.

## Authors' contributions

JMK, LBH, and SLB prepared, read, and approved the final manuscript.
